# Frailty Is an Independent Marker of Post‐Operative Mortality Following Colorectal Cancer Resection Surgery in Older Adults

**DOI:** 10.1002/jso.28137

**Published:** 2025-05-15

**Authors:** Maria Normann, Niklas Ekerstad, Eva Angenete, Mattias Prytz

**Affiliations:** ^1^ Department of Surgery, Institute of Clinical Sciences, Sahlgrenska Academy University of Gothenburg Gothenburg Sweden; ^2^ Department of Surgery Region Västra Götaland, NU‐Hospital Group Trollhättan Sweden; ^3^ Department of Health, Medicine and Caring Sciences Linköping University Linköping Sweden; ^4^ Department of Research and Development Region Västra Götaland, NU‐Hospital Group Trollhättan Sweden; ^5^ Department of Surgery, SSORG – Scandinavian Surgical Outcomes Research Group, Institute of Clinical Sciences, Sahlgrenska Academy University of Gothenburg Gothenburg Sweden; ^6^ Department of Surgery Region Västra Götaland, Sahlgrenska University Hospital Gothenburg Sweden

**Keywords:** colorectal neoplasms, elderly, surgery frailty

## Abstract

**Background and Objectives:**

Frailty is a prognostic factor of post‐operative death and complications following colorectal cancer surgery. Frailty assessment is not routinely performed, hence, the prevalence is unknown. The aim of this study was to establish the presence of frailty in patients aged ≥ 70 years, and to analyse differences in post‐operative outcome comparing frail and non‐frail elderly patients.

**Method:**

Data of patients aged ≥ 70 years who underwent colorectal cancer surgery during 2016–2020 were retrospectively obtained from the Swedish Colorectal Cancer Registry. A cohort of 500 patients was assessed for frailty using the Clinical Frailty Scale (CFS‐9). Post‐operative mortality rates, complications, readmissions, and length of stay (LOS) were compared between frail and non‐frail patients.

**Results:**

The prevalence of frailty (CFS‐9 score ≥ 4) was 56%. The 90‐day mortality rate was higher in frail patients (OR 4.97 [95% CI 1.06–23.28], *p* 0.042), as well as 1‐year mortality (OR 4.39 [95% CI 1.86–10.34], *p* 0.0007). Frail patients had longer post‐operative LOS (7.63 vs. 11.0 days, *p* < 0.001), were more often treated in ICU and more often discharged to a nursing home.

**Conclusion:**

Frailty is a common condition in patients ≥ 70 years and a significant risk factor of post‐operative mortality and morbidity.

**Abbreviations**
ADL: activities of daily life
ASA: American Society of Anaesthesiologists
CFS‐9: Clinical Frailty Scale‐9
CGA: comprehensive geriatric assessment
CI: confidence interval
CRC: colorectal cancer
ICU: intensive care unit
LOS: length of stay
OR: odds ratio
SCRCR: Swedish Colorectal Cancer Registry
SD: standard deviation
TNM: tumour nodemetastasis
VGR: Region Västra Götaland

## 1 Introduction

### 1.1 Background

The greater part of newly diagnosed colorectal cancer cases are amongst older adults (≥ 70 years), and it is also a significant cause of death in this age group [1, 2, 3]. Although the treatment of colorectal cancer has improved, rendering better overall post‐operative survival, this is only partly the case amongst older patients. Especially, the immediate post‐operative mortality and morbidity are increased amongst older adults [4, 5, 6, 7, 8]. We have previously shown that patients ≥ 70 years have an increased 90‐day mortality rate post colorectal cancer surgery compared to younger individuals [9].

Although increased chronological age is a negative prognostic factor, there is a vast inter‐individual diversity within an age group [1, 10, 11]. Frailty is a clinical syndrome which can be used as a marker of biological age [12, 13]. Frailty has previously been described as an independent risk factor associated with adverse effects such as increased post‐operative mortality, functional dependence, readmission to hospital and need of residential care [5, 14, 15, 16]. Frail elderly patients are a high‐risk group also in colorectal cancer surgery [10, 11, 17, 18] and that frailty is more strongly associated with negative outcome than age [5].

One of the most commonly used screening instruments for frailty is the Clinical Frailty Scale (CFS‐9), which can be assessed during a routine patient visit [12, 14, 19, 20, 21, 22]. CFS‐9 is developed from the accumulation of deficits model of frailty and is based on the care‐takers evaluation of the patient's level of fitness, presence of disease symptoms, the degree of dependence from others and of cognitive function [23, 24]. The instrument is a 9‐item tool where patients who are assessed as CFS 4–8 are considered frail [25]. The assessment is intended to be done during a clinical interview, but recent studies have shown a reliable correspondence between standard evaluation and retrospective valuation through medical chart review [26, 27, 28, 29]. The aim of this study was to investigate whether frailty is an explanatory factor of the poorer post‐operative outcome in older adults in Sweden.

### 1.2 Objectives

The aim of this study was to evaluate if frail patients in this cohort had poorer post‐operative outcome following colorectal cancer resection surgery compared to non‐frail. The hypothesis was that frailty is a risk factor for post‐operative mortality and morbidity following CRC resection surgery in older adults. The primary endpoint was differences in 90‐day overall survival, secondary endpoints were post‐operative complications, reoperations, intensive care unit (ICU) care, post‐operative hospital length of stay (LOS), readmissions within 30 days post‐surgery, discharge destination (own housing, nursing facility) and all cause 1‐year mortality.

## 2 Methods

### 2.1 Study Design, Setting and Participants

This is a retrospective, registry‐based, cohort study performed in a Swedish Region of 1.7 million inhabitants during January 2016 to June 2020. The study population included all patients aged ≥ 70 surgically treated for colorectal cancer in Region Västra Götaland (VGR) during the stated period (*n* = 2378). The study was approved by the Swedish Ethical Review Authority (Dnr 2021‐02099 and Dnr 2022‐03759‐02).

A sample size calculation based on a 5% difference in 90‐day mortality between non‐frail (CFS‐9 1–3) and frail (CFS‐9 ≥ 4) patients was conducted. The presence of frailty in the current material was 56% and 90‐day mortality in the non‐frail group was assumed to be 1.5%. To detect a difference with 80% power and at a significance level of 0.05, a total of 480 patients was needed.

### 2.2 Variables and Data Sources

A cohort of 500 patients was randomly selected from all patients diagnosed and operated for colorectal cancer during 2016 and 2020 in the studied region and assigned a frailty score according to CFS‐9, through retrospective chart review. The medical charts of these patients were analysed regarding previous medical history and comorbidities, current medications, recent visits to the emergency department or hospitalisation episodes, recent investigations, and physical examinations. Background information such as housing situation, social history, access to formal and informal supports, use of gait aid, cognitive status and activities of daily life (ADL) function were also assessed through the medical records. These factors were used to assign patients a CFS‐score of 1–9 and the available information was sufficient to issue a CFS‐score for all subjects in the cohort.

Baseline data for all subjects were retrospectively obtained from the Swedish Colorectal Cancer Registry (SCRCR), a nationwide registry of patients with colorectal cancer, with high coverage (> 98%) [30]. The following baseline variables known to influence death after colorectal cancer surgery were obtained from the registry: age, sex, tumour location, stage according to TNM, American Society of Anaesthesiologists (ASA) classification and elective versus urgent surgery. It is known that elderly patients are more often treated with urgent surgical resection compared to younger individuals [4, 6], hence, this variable was treated as a mediator rather than a confounder and was not adjusted for. A Directed Acyclic Graph (DAG) showing the relationship between the variables and the exposure (frailty) and outcome (mortality) can be found in the Supporting Information.

### 2.3 Statistical Methods

Analyses of dichotomous outcome variables were made through logistic regression and Fisher's exact test and presented as odds ratio (OR) with 95% confidence interval (CI). Adjusted analyses were made through multivariable logistic regression and presented as OR with 95% CI. Post‐operative hospital LOS was analysed using Fisher's non‐parametric permutation test and multiple logistic regression, presented as mean difference with 95% CI. For all models, the independent variable CFS‐9 was treated as follows: the subgroup of non‐frail patients (CFS 1–3) served as reference group and was compared with each step from 4 to 9. CFS‐9 was also analysed as a continuous variable presenting the risk when increasing CFS‐9 one step. A dichotomised analysis between non‐frail patients (CFS 1–3) and frail patients (CFS ≥ 4) was also performed. All tests were two‐tailed and conducted at 0.05 significance level.

## 3 Results

### 3.1 Patient Characteristics

Baseline characteristics of the cohort are described in Table 1. The prevalence of frailty in the entire cohort was 56%. Non‐frail patients were significantly younger than frail patients (76.2 vs. 80.0, *p* < 0.001), the sex distribution was similar but there was a slight overrepresentation of women in the frail group. There were more ASA III–IV individuals in the frail group compared to the non‐frail group, and non‐frail patients were treated for more advanced disease and more often went through elective surgery.

**Table 1:** Patient characteristics for the randomly selected cohort (*n* = 500), tabulated by CFS‐9 (< 4 vs. ≥ 4).

Variables		All subjects (*n* = 500)	Non‐frail, CFS‐9 1–3 (*n* = 220)	Frail, CFS‐9 ≥ 4 (*n* = 280)
Age		78.4	76.2 (±4.8)	80.0 (±6.0)
Sex				
	Women	249	101 (45.9)	148 (52.9)
	Men	251	119 (54.1)	132 (47.1)
CFS‐9 score	1	20	20 (9.1)	—
	2	24	24 (10.9)	—
	3	176	176 (80.0)	—
	4	168	—	168 (60.0)
	5	63	—	63 (22.5)
	6	34	—	34 (12.1)
	7	5	—	5 (1.8)
	8	0	—	0
	9	5	—	5 (1.8)
ASA classification				
	ASA 1–2	281	180 (81.8)	101 (36.1)
	ASA 3	186	34 (15.4)	152 (54.3)
	ASA 4–5	20	1 (0.5)	19 (6.8)
	Missing	13	5 (2.3)	8 (2.9)
Surgical prioritisation				
	Elective	439	200 (90.9)	239 (85.4)
	Urgent	61	20 (9.1)	41 (14.6)
Clinical stage (cTNM)				
	I	187	82 (37.3)	105 (37.5)
	II	123	45 (20.5)	78 (27.9)
	III	152	79 (34.5)	73 (26.1)
	IV	40	17 (7.7)	23 (8.2)
	Missing	1	—	1 (0.4)
Tumour location				
	Colon	251	119 (54.1)	132 (47.1)
	Rectum	249	101 (45.9)	148 (52.9)

*Note:* Baseline variables presented for all subjects. The values were expressed as mean (SD) and number (%).

Abbreviations: ASA, American Society of Anaesthesiologists; CFS‐9, Clinical Frailty Scale‐9; SD, standard deviation; TNM, tumour node metastasis.

### 3.2 Outcome Variables

**Figure 1:**
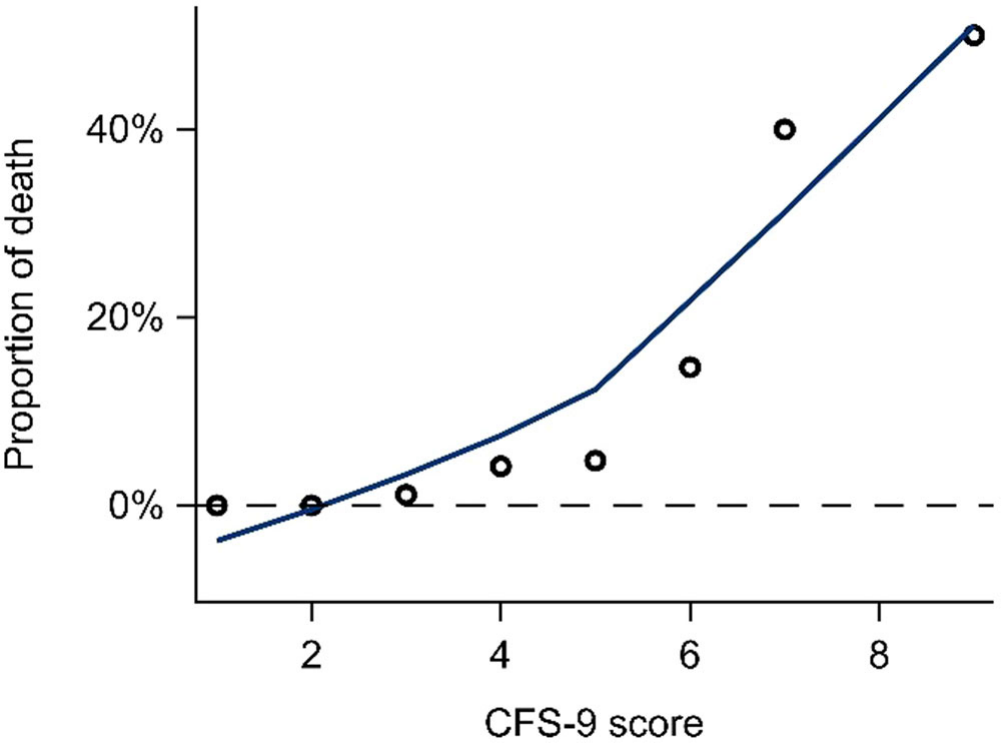
Proportion of death within 90 days for each step of CFS‐9 score.

Multivariable analyses showed a significantly increased 90‐day mortality risk for frail patients and there was an evident risk increase for each step of the CFS (Table 2 and Figure 1). This was also confirmed in analyses using CFS as a continuous variable (data not shown). Further, an increased risk of post‐operative surgical complications for frail patients was revealed, however, this was not shown in analyses of overall complications (Table 3). It was more common amongst frail patients to be treated in ICU postoperatively compared to non‐frail patients, when the CFS was analysed as a continuous variable (1.38 [1.08–1.78], *p* 0.011). Post‐operative LOS compared between non‐frail and frail patients was significantly longer (7.63 vs. 11.0 days, *p* < 0.001). Frail patients were also more likely to be discharged to a nursing home compared to non‐frail patients (Table 3). One‐year mortality was significantly increased for all stages of frailty. There was no significant increased risk for reoperations or readmissions within 30 days for frail patients.

**Table 2:** Multivariable analyses of the primary endpoint, 90‐day mortality following colorectal cancer resection surgery.

	90‐day mortality		
CFS‐9 score	*n* of events (%)	OR (95% CI)	*p*
CFS 1–3	2 (0.9)	1.00	
CFS 4	7 (4.2)	2.94 (0.56–15.43)	0.20
CFS 5	3 (4.8)	2.59 (0.35–19.06)	0.35
CFS 6	5 (14.7)	8.18 (1.26–53.22)	**0.028**
CFS 7	2 (40.0)	28.61 (2.16–378.58)	**0.011**
CFS 9	5 (50.0)	58.25 (7.28–465.80)	**0.0001**

*Note:* Frail patients categorised according to CFS‐9 scale and analysed in comparison to non‐frail patients (CFS 1–3). Variables presented as *n* (%). Multivariable analyses were made using a multivariable logistic regression mode, adjusted for age, sex, ASA‐classification, tumour stage, and tumour location, values expressed as OR (95% CI). Bold values indicate statistical significance.

Abbreviations: ASA, American Society of Anaesthesiologists; CFS, Clinical Frailty Scale; CI, confidence interval, OR, odds ratio.

**Table 3:** Multivariable analyses of primary and secondary endpoints, dichotomised groups (non‐frail 1–3 vs. frail 4–9).

	*n* (%) of events			
Variables	Non‐frail	Frail	OR (95% CI)	*p*
90‐day mortality	2 (0.9)	22 (7.9)	4.97 (1.06–23.28)	**0.042**
Post‐operative complications	72 (33.2)	114 (41.0)	1.53 (0.98–2.39)	0.062
Post‐operative surgical complications	22 (10.0)	54 (19.3)	2.86 (1.51–5.40)	**0.0012**
Reoperations	17 (7.8)	34 (12.2)	1.60 (0.78–3.28)	0.20
Post‐operative ICU care	8 (3.7)	27 (9.7)	2.32 (0.89–6.08)	0.087
Readmissions	20 (9.2)	33 (11.9)	1.28 (0.64–2.57)	0.49
Discharge destination (home vs. nursing facility)	215 (99.1)	253 (94.1)	0.16 (0.03–0.78)	**0.023**
12 months mortality	8 (3.6)	45 (16.1)	4.39 (1.86–10.34)	**0.0007**

*Note:* Variables presented as *n* (%). Multivariable analyses were made using a multivariable logistic regression mode, adjusted for age, sex, ASA‐classification, tumour stage, and tumour location, values expressed as OR (95% CI). Bold values indicate statistical significance.

Abbreviations: ASA, American Society of Anaesthesiologists; CFS, Clinical Frailty Scale; CI, confidence interval, OR, odds ratio.

## 4 Discussion

Our study confirms that frail patients have poorer post‐operative outcome following colorectal cancer surgery than non‐frail patients, as shown by our primary outcome 90‐day mortality. Although the cohort size is limited, our data are in accordance with previously published data, but to our knowledge, this has not previously been studied in a Swedish setting. Whether the increased short‐term mortality in this group could be mitigated by interventions, such as comprehensive geriatric assessment (CGA), prior to surgery is to date unknown. However, in other clinical contexts, such as hip fracture care, it has been shown to be beneficial [31].

In addition, 1‐year mortality was also more pronounced in the frail group. If this is directly related to the colorectal cancer surgery or to other comorbidities or events, cannot be elucidated from our data. Still, it is of interest to note that frail patients operated for colorectal cancer have a shorter life expectancy than non‐frail. Another aspect is the selection of patients before surgery, perhaps our results would be even more pronounced if more frail patients were selected for surgery. The selection of patients to surgery is presumably already today influenced by the surgeons' overall assessments of patients' level of function, but not using standardised frailty instruments. Incorporating the use of CFS, in addition to age and comorbidities, in clinical decision making enables identification of individuals of increased risk and of reduced reserves.

Urgent surgery was more common among frail patients, as could be expected since elderly patients are more often treated in an emergency setting [9]. This was also the reason not to adjust for urgent surgery in the multivariable analyses as it was considered a mediator rather than a confounder. Patients operated in an urgent setting have more complications and worse outcome and urgent procedures should be prevented if possible [8, 32]

Somewhat surprisingly, we found no significant differences in overall complications between the two groups. This could be a matter of sample size but might also be due to an underreported registration of this variable in the registry [33]. We did find a tendency towards more ICU care in the frail group, indicating a more complicated post‐operative period. We also found a difference in surgical complications, and notably the registration of surgical complications is known to be more accurately reported in the registry [33]. There were no differences in the rate of readmission within 30 days post‐surgery. However, as frail patients had a longer LOS after surgery and were more often referred to a nursing home when discharged, these numbers are difficult to interpret. The LOS in hospital in this cohort of patients was generally longer than what is common within an ERAS concept. The reasons behind this are not further explored within the scope of this article, but a contributing factor could be the extended need of home care services in the frail group, resulting in longer stay in hospital. To our knowledge, all hospitals except one in the present study apply an ERAS‐concept in routine colorectal cancer surgery, though our insight in each centra's adherence to the protocol is limited.

A strength of our study is the completely random selection of patients from a quality register with high coverage, including all patients treated for colorectal cancer during the study period. This reduces selection bias and increases the generalisability of our data. The use of a well‐validated instrument to assess frailty, evaluated by the same researcher, increases the internal validity. However, the retrospective assessment could be considered a limitation. In a previous study performing retrospectively CFS screening through medical charts, a geriatric nurse had done an interview with the patients, ensuring relevant data to perform the screening being available. This study showed high accordance between prospectively and retrospectively assigned frailty scores [26]. Other studies have also described good accuracy of CFS scores generated through medical charts, though with moderately higher prospectively gained scoring [27, 28]. In the current study, all reviews were made by one assessor with previous experience in performing retrospective frailty assessment using CFS‐9. The reviewer had received thorough training in using the CFS‐9 scale by one of the leading experts on CFS‐assessment in Sweden [34]. As the use CFS‐9 in retrospectively assessing frailty has previously been described and validated, and as the reviewer in our study did not have a role in the interpretation of data, another independent reviewer was not deemed necessary [26, 27, 29].

A limitation is that we do not have data on selection to surgery, thus the differences in tumour stage in frail versus non‐frail patients could be due to selection bias. Whether this influences the results is uncertain, but most probably the data are representative in a larger clinical context. The lack of detailed information on comorbidity is a limitation, but we have tried to mitigate this by using the ASA classification. Another limitation is the relatively small study population in this material, as some events were few and for some of the secondary outcomes, the numbers were probably too small to reach statistical significance. Still, our power calculation indicated that for the primary endpoint the study was sufficiently dimensioned.

The insights from this study further elucidate the increased risk of performing surgery on frail elderly patients. Since we know that the elderly population is expanding, future research needs to further explore how to make appropriate decisions regarding when to operate and when to refrain from surgery. To improve decision making, focus needs to be on post‐operative outcome in terms of quality of life and functional status, as well as complications and mortality. In addition, frailty is a dynamic condition where interventions such as comprehensive geriatric assessment and care (CGA) have been proven beneficial in other clinical contexts. In an ongoing randomised controlled trial led by the authors of this article, the effects of CGA and care prior to colorectal cancer resection surgery are being evaluated. The results of this trial, in addition to the findings in this study, could have importance in the future care of frail elderly patients diagnosed with colon and rectal cancer.

## 5 Conclusion

Frailty is a common condition amongst patients ≥ 70 years with colorectal cancer and the presence of frailty increases the risk of post‐operative mortality both in short‐ and long‐term. Frail elderly patients would presumably benefit from an individually targeted treatment with respect of their high‐risk situation.

## Disclosure

The funding parties have no role in the design of the study or collection, analysis, or interpretation of data, neither in writing of the manuscript.

## Synopsis

Frailty is a common condition in patients aged ≥ 70 years and is associated with poorer post‐operative outcome after colorectal cancer surgery.

## Supporting information

DAG.

## Data Availability

The data that support the findings of this study are available from the corresponding author, (M.N.), upon reasonable request.
