# Supplementary figures and images for: Frailty Is an Independent Marker of Post‐Operative Mortality Following Colorectal Cancer Resection Surgery in Older Adults
Source: J Surg Oncol. 2025 May 15;132(1):198–204. doi: 10.1002/jso.28137 (PMC12311407; doi:10.1002/jso.28137)

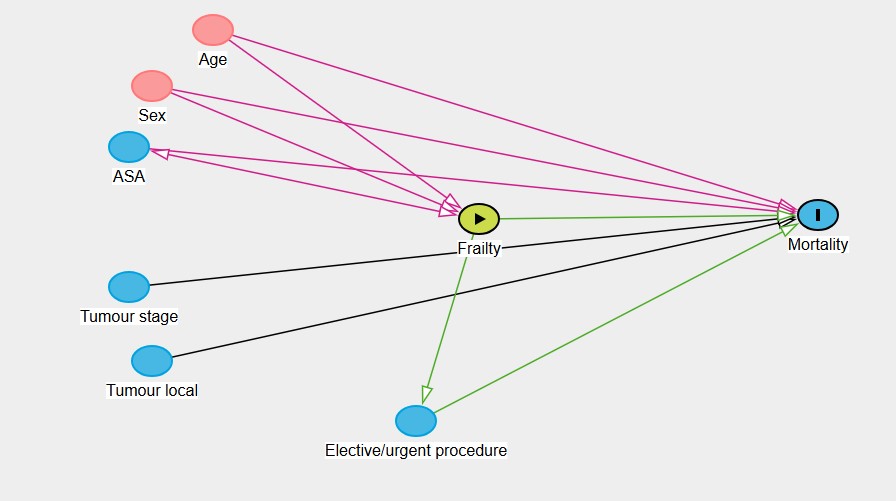

Supplement: Supplementary file 1 — DAG. [file JSO-132-198-s001.jpg]
